# Network Assemblages of Elevational Niche‐Associated Diversity in Fijian Native Bees

**DOI:** 10.1002/ece3.71073

**Published:** 2025-03-02

**Authors:** Patricia S. Slattery, James B. Dorey, Cale S. Matthews, Justin L. Holder, Olivia K. Davies, Mark I. Stevens, Michael P. Schwarz, Carmen R. B. da Silva

**Affiliations:** ^1^ College of Science and Engineering Flinders University Bedford Park South Australia Australia; ^2^ Environmental Futures Research Centre, School of Science University of Wollongong Wollongong New South Wales Australia; ^3^ Australian Catholic University Fitzroy Victoria Australia; ^4^ Earth and Biological Sciences South Australian Museum Adelaide South Australia Australia; ^5^ School of Biological Sciences University of Adelaide Adelaide South Australia Australia; ^6^ School of Natural Sciences Macquarie University North Ryde New South Wales Australia

**Keywords:** biogeography, competition, *Lasioglossum*, nestedness, network analyses, niche

## Abstract

Species assemblages constrained by ecological and evolutionary processes (and the interactions between them) are vulnerable to changes in their environment. Network analyses do not explicitly build in phylogenetic histories when exploring how they are assembled, yet they can be a critical source of information for understanding how and when species may be incorporated into ecological webs. Recent studies have revealed unexpected species diversity in a monophyletic clade of native Fijian bees in the subgenus *Lasioglossum* (*Homalictus*). These bees have undergone a remarkable and recent radiation with evidence for phylogenetic conservatism in elevational niches and physiological traits. Here we use bipartite network analyses, as an adjunct to phylogenetic analyses, to further inform likely ancestral elevations for these bees and to explore patterns in how they have occupied other elevational niches. Our approach is novel in that we categorize elevation into bands that are then treated as the lower hierarchical level onto which we map individual bee species. These analyses support earlier inferences that highland elevations (or the climates that correspond to them) are ancestral niches and that barriers to occupation of lower elevations are significant. In addition, we provide important insights into co‐occupancy of elevational niches and whether competition occurs for these niches. Our results suggest convergences in niche expansion and a lack of competitive exclusion for those specific niches, but a strong extinction risk for loss of current elevation‐related niches.

## Graphical abstract

Using network analyses, this paper demonstrates the aggregated distribution of Fijian *Lasioglossum* (*Homalictus*) bees in elevational niches. Combining the nestedness explored in this study with previous research, we highlight the conservatism in these bees and discuss their vulnerability as a result.

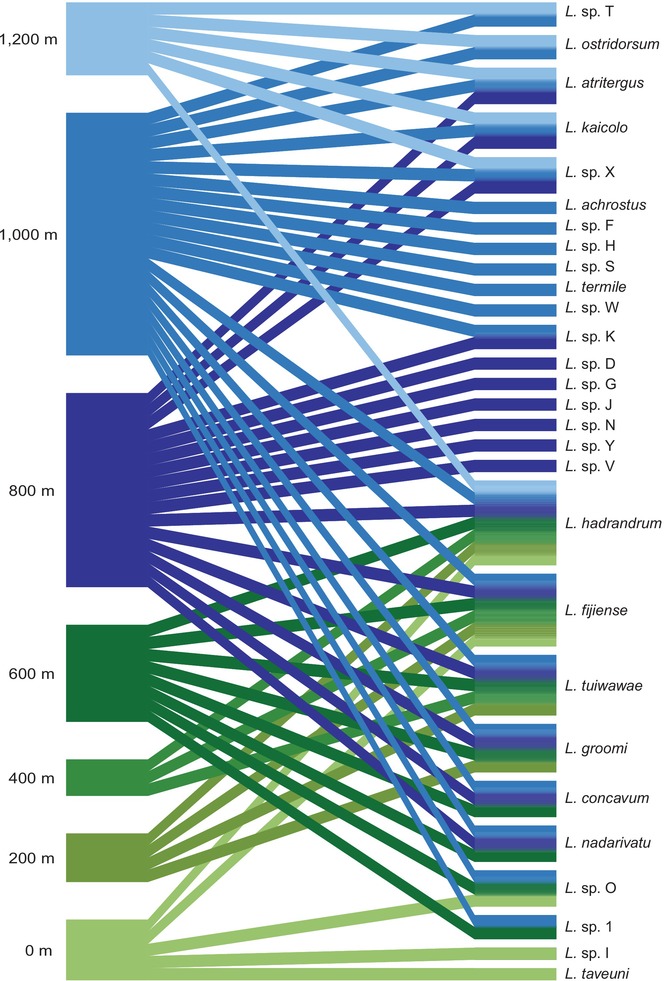

## 1 Introduction

Understanding the drivers of species distributions across space is a key goal in evolutionary ecology (Baselga et al. 2012; Comte et al. 2014; Kellermann et al. 2012; Mayr and O'Hara 1986). Patterns in species' niches (e.g., climatic niche) across space and time are frequently examined using phylogenetic niche conservatism analyses (Cadena and Céspedes 2020; da Silva et al. 2024; Dorey et al. 2019; Patton and Smith 1992; Wiens et al. 2019). Species composition within and across sites can be examined using measures of—and—phylogenetic diversity, respectively and trends can be explored using analytical tools such as Mantel tests (Faith 1992; Webb et al. 2002, 2008). However, network analyses provide an additional and complementary toolbox for exploring such patterns, using visual aids as well as parameters such as nestedness, extinction slopes and measures of competition for niche occupancy (Dormann et al. 2008, 2009).

Bipartite network analyses in ecology have been used to explore how different hierarchical levels may be connected and how those connections could have been shaped by ecological or evolutionary processes (Delmas et al. 2019; Dormann et al. 2008). Studies have focused on systems such as predator–prey, host–parasite and plant–pollinator networks (Dursahinhan et al. 2023; Gelmi‐Candusso et al. 2023; Slattery et al. 2023; Suzuki et al. 2023) to explore issues such as whether higher‐level entities, like pollinators, compete with each other for access to lower‐level resources, such as flowering plant species (Hayes et al. 2019; Martínez‐López et al. 2024; Proesmans et al. 2021). However, partitioning in the use of lower‐level elements by higher‐level elements can be driven by many other kinds of interactions, such as convergence in resource exploitation, adaptive radiation and retention of ancestral traits.

Historically, network analyses have been used to explore species assembly in island biogeography (e.g., Kougioumoutzis et al. 2014; Trøjelsgaard and Olesen 2013). A classic scenario imagines a mainland with high species diversity where some species can colonise nearby islands, which in turn provide colonisers for successively remote islands. In the absence of within‐island speciation, such a situation should lead to nested species compositions, with the more remote islands having smaller and smaller subsets of the mainland species. Intriguingly, Marini et al. (2019) recently outlined possibilities for using bipartite networks to explore species–habitat relationships where different habitats might be contiguous and without hard borders, such as across elevational gradients. However, problems might arise if dispersal distances are large compared to habitat patch size, or if spatial heterogeneity in habitats is high (Marini et al. 2019).

Nestedness is a key attribute of networks with particular relevance to conservation biology. In a tightly nested system, the loss of one lower‐level element (e.g., a mainland) will have greater extinction consequences than the loss of higher‐level elements (e.g., islands), and the extent of this will depend on the degree of nesting. In terms of conservation, it becomes important to understand if some higher‐ or lower‐nested elements have different susceptibilities to being lost. We might conceptually compare an ancestral niche, which holds the greatest diversity of a particular taxonomic group, to a mainland. In this scenario, any process that threatens an ancestral niche would have a greater impact than one that threatens more derived niches, where taxa may have diversified more recently and not accumulated the same richness.

The Fijian archipelago provides an ideal system for examining relationships between species and their elevational niches with network analyses. Fiji consists of coastal lowland habitat, which comprises 78% of the forests in Fiji (SPREP 2013), and steep mountains with cloud forest peaks. The first taxonomic revision of native Fijian bees (Michener 1979) recognized only four species in the subgenus *Lasioglossum* (*Homalictus*) (Hymenoptera: Halictidae), three of which were restricted to elevations above 800 m asl (above sea level). Using a combination of morphology and DNA sequence data, Dorey et al. (2019) increased this number to 13 species; however, species diversity of this monophyletic clade (Ibalim et al. 2020) is likely to be in excess of 22 (Dorey et al. 2020). Species richness increases with elevation, where most species only occur over 800 m asl (da Silva et al. 2024), and many species are restricted to single mountain peaks (Dorey et al. 2019, 2020). These species likely evolved through interactions between past climate cycles and phylogenetic conservatism of cool niches (Dorey et al. 2020), which are currently associated with high elevation ‘sky islands’ (da Silva et al. 2024). Restriction of species to elevational niches raises the question of whether ‘sky islands’ could be figuratively treated as islands in biogeographic network analyses, allowing concepts like ancestral niches, barriers to dispersal, competitive exclusion and extinction vulnerability to be explored using network statistics.

Here we take a bipartite network approach to explore how native Fijian *Lasioglossum* (*Homalictus*) diversity is assembled across elevational niches. We aim to determine whether such an approach can yield insights into the nestedness and associated extinction proneness of these elevational niches. When occupation and dispersion within an ecosystem are constrained by ecological and evolutionary processes, species assemblages and consequent nestedness measures would reflect this. Under this assumption, the greatest diversity should be found in (and largely restricted to) ancestral niches. Previous research suggests that species richness is greatest in the highlands for these bees and that restrictions to dispersal are due to both physiology and environment (da Silva et al. 2024; Dorey et al. 2020). Therefore, we hypothesise that species at higher elevations, where diversity is greatest, will exhibit more nestedness and stronger associated extinction risks than lowland species. This will be reflected in both higher nestedness and extinction slope estimates in our bipartite analyses.

## 2 Methods

### 2.1 Bee Sampling

**FIGURE 1:**
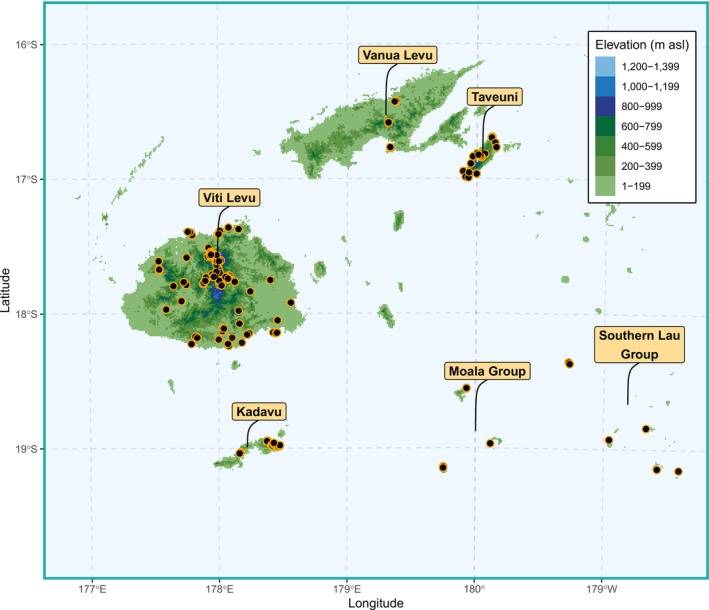
Map of collecting localities of *Lasioglossum* (*Homalictus*) in the Fijian archipelago. Specimens were collected from 2010 to 2019.

We sampled 2190 *Lasioglossum* (*Homalictus*) specimens throughout the Fijian archipelago between 2010 and 2019, including the main islands of Viti Levu, Vanua Levu, Taveuni, and Kadavu, as well as multiple islands within the Lau group (Figure 1). Sampling localities ranged from sea level to 1324 m asl. Samples were obtained by sweep‐netting flowering plants and digging up nesting aggregations, which mostly occurred in bare soil. Latitude, longitude, and elevation for each sample were recorded using a Garmin 550 GPS (Garmin Ltd., USA) and confirmed using Google Earth. All samples were preserved in > 98% ethanol and stored at < 4°C for later DNA sequencing.

### 2.2 Species Delimitation

Species were identified via morphological descriptions and from a ≥ 630 bp fragment of mitochondrial DNA (cytochrome *c* oxidase subunit I), according to descriptions by Dorey et al. (2019). We combined our new (2018–19) sequences with those available on Dryad from previous studies (Dorey et al. 2020; Groom et al. 2014). All samples had DNA extracted from a single hind leg, with samples collected 2010–2014 amplified at the Canadian Centre for DNA Barcoding (CCDB) at the Biodiversity Institute of Ontario (Ivanova et al. 2006). For specimens collected 2015–2018, DNA extraction and PCR were completed at the South Australian Regional Facility for Molecular Ecology and Evolution, and methods are described in detail in previous studies (Dorey et al. 2019, 2020; Groom et al. 2013). Specimens collected in 2019 were sent to the Centre for Biodiversity Genomics, where DNA was extracted and sequenced using the SEQUEL platform, as described by Hebert et al. (2018). We then checked these sequences against the National Center for Biotechnology Information (NCBI) BLAST to remove non‐target sequences.

### 2.3 Elevational Bands

As noted by Michener (1979), of the four Fijian *Lasioglossum* (*Homalictus*) species known at that time, three were limited to elevations above 800 m asl. Dorey et al. (2020) showed that of the 22 species they recognised from morphological and DNA sequences, 18 had median elevational bands above 800 m asl and many species were short‐range endemics recorded from very narrow elevational ranges or just single localities. We categorised sampling localities into seven 200 m elevational niches (0–199, 200–399 … 1200–139 m asl) and recorded the number of species falling into each category (Supplementary—S1).

### 2.4 Bipartite and Network Analyses

Island biogeographic network analyses typically treat mainlands or islands as sampling units for exploring distribution nestedness, and these can then be used to infer ancestral species ranges and dispersal patterns (Dennis et al. 2012; Marini et al. 2019; Ulrich et al. 2009). For our analyses, we treated each elevational band as discrete but contiguous with both lower and higher elevations. Strictly speaking, these niches do not represent true islands since our categories are somewhat arbitrary and, instead of a discrete barrier—such as a water expanse, we rely on capturing gradual changes in niche. However, Dorey et al. (2020) showed that the distributions of most Fijian *Lasioglossum* (*Homalictus*) species are constrained by elevational niche, with one major niche below 800 m asl and several above. Hence, we can think of elevation as a niche barrier that limits dispersal and use ancestral high‐elevational niches (da Silva et al. 2024; Dorey et al. 2020) as a proxy for ‘mainlands’ in a classic biogeographic network analysis. However, the interpretation of network analyses using elevational niches as ‘islands’ needs to be cautiously interpreted, keeping in mind that they may represent weaker delineations, similar to different habitat types within a single landscape (Marini et al. 2019).

Network analyses were carried out in R (v4.3.3; R Core Team 2024) and RStudio (v2024.04.1 + 748; RStudio Team 2024), using the package *bipartite* (v2.19; Dormann et al. 2008). For these analyses, we used unweighted binary counts (i.e., zero or one) for each combination of species and elevational niche (a weighted nestedness matrix is available as Supplementary—S1). Network structure was visually explored using the *bipartite* functions, ‘visweb’ and ‘plotweb’, and network statistics were calculated using ‘networklevel’ (Table 1). Where appropriate, the statistical significances of network parameters were assessed using simulated null models in the package *vegan* (v2.6–4; Oksanen et al. 2024). For this, we used the ‘r00’ null model (run for 1000 permutations) which retains the absolute number of filled (presence) cells in the distribution matrix but allocates ± cells randomly across the matrix. We then calculated cumulative species loss by the removal of elevational niches. Species had to be present only in the elevational niche removed or any higher elevations. These losses were plotted with the package *ggplot2* (v3.5; Wickham 2016).

**TABLE 1:** Network statistics based on binary species/elevation Fijian *Lasioglossum* (*Homalictus*) occurrence records.

Statistic	Null model	Observed value	Interpretation	Definition
Nestedness	52.930	17.732***	Nestedness is significantly greater than randomised occurrences	Nestedness is a measure of the robustness of the network, based on how well lower richness nodes form a subset of higher richness nodes
NODF	34.428	55.9130***	Nestedness across elevational niches is significantly greater than randomised occurrences	NODF is a more robust measure of nestedness based on overlap and decreasing fill, taking into consideration both if the total number of filled cells differs at either level, and if presences in less filled columns/rows of the matrix coincide with those in more filled columns/rows
Togetherness (species)	0.181	0.206^NS^	Co‐presences and co‐absences of elevational niches by pairs of species are not different from random co‐occupancies	The proportion of co‐absences and co‐absences in the matrix, in this case defined as two bee species next to each other in the matrix that both occur at the same elevation and then both don't occur at the next elevation
C‐score (species)	0.485	0.249***	Species are aggregating within many of the same elevational niches	Based on a checkerboard pattern in the matrix, the proportion of species that occurred in one elevational band, but not the next, with the next species in the matrix displaying the same pattern
V‐ratio (species)	0.994	8.027***	Evidence for positive aggregation of species within elevational bands	The variance ratio of species numbers to individuals within species. Essentially, a measure of species association, testing if it is more or less likely to find one species if there is another species present
Extinction slope (species)	2.392	1.888***	Random loss of elevational bands will lead to higher rates of species extinctions than for randomised occurrences	Used to explore patterns of extinction of level ‘A’ based on the removal of sequences of ‘B’ at the opposite level. The higher the slope estimate, the less level ‘A’ will be affected by the removal of level ‘B’

*Note:* Parameters and guides to their interpretation are described in Dormann et al. (2009) and references therein. The null models were based on the average of ‘r00’, which maintains the number of filled cells in the distribution matrix but allocates them randomly across the matrix for each permutation. Significance indicated as: NS no significant difference between null model and observed value at *α* = 0.05 level, *** < 0.001.

## 3 Results

**FIGURE 2:**
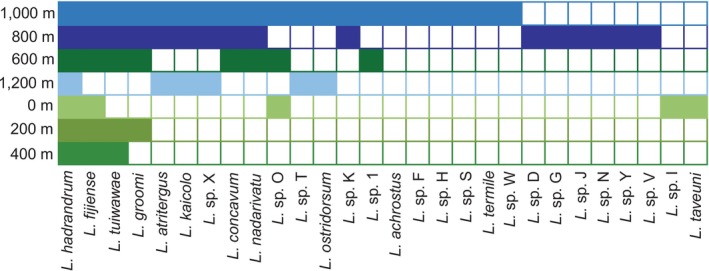
Nested matrix depicting species occupancy across the elevational gradient, separated into 200 m asl elevational niches. Solid cells in the matrix denote the presence of Fijian *Lasioglossum* (*Homalictus*) species within that elevational niche. Blue colours correspond to highland (> 800 m asl) elevational niches, and green colours correspond to < 800 m asl elevational niches.

**FIGURE 3:**
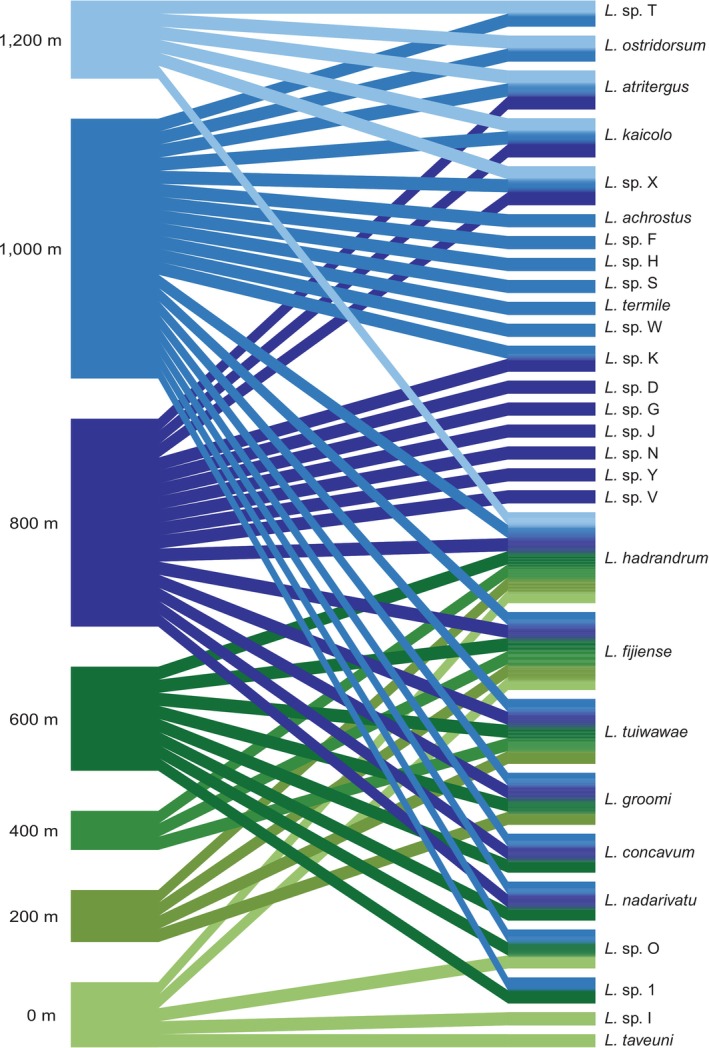
Bipartite plot linking elevational niches (left) to Fijian *Lasioglossum* (*Homalictus*) species' presence across the elevational gradient. Width of the bands representing the elevational niche implies number of bee species present in that niche, while width of bands for each bee species indicates their elevational niche distribution. Blue colours correspond to highland (> 800 m asl) elevational niches, and the green represent non‐highland (< 800 m asl) elevational niches.

Fijian *Lasioglossum (Homalictus)* species richness and diversity are concentrated at elevations between 800 and 1324 m asl, the latter elevation being the peak of Fiji's tallest mountain, Tomanivi (Figures 2 and 3). The highest species richness (*n* = 20) was found in the 1000 m asl band and the second greatest (*n* = 16) in the 800 m asl band. While the 1200 m asl band contained only six species and corresponded to only Mount Tomanivi (and its immediate flanks) or the Rairaimatoku plateau, both of which are on the main island of Viti Levu.

Both of our suggested values of nestedness (null = 53, observed = 17, *p* < 0.001; Table 1) and NODF (nestedness based on overlap and decreasing fill; null = 34, observed = 56, *p* < 0.001; Table 1) supported this, with lower elevational niches forming a subset of the higher elevation niches (Figure 2). These nestedness statistics were significantly different from ‘oecosimu’ simulations of the null model (random occupancy of elevational niches) (Table 1). The bipartite network further demonstrates the strong pattern of species distributions coinciding with elevational niches (Figure 3). Of the 28 species included, 18 of those species are restricted to the highlands (> 800 m asl), and only two species are restricted to < 800 m asl (Figure 2).

Network analyses based on the binary occupancy data provided statistical support for the aggregation of more species in the highlands (Table 1). The togetherness score of this network was low and not significant (null = 0.18, observed = 0.21, *p* > 0.05; Table 1), indicating that the co‐presence and co‐absence of species in elevational niches were not significantly different from randomised data. A significantly lower C‐score (null = 0.46, observed = 0.25, *p* < 0.001; Table 1) from the null model demonstrates that species are occupying many of the same elevational niches (i.e., there is no evidence for competition of elevational niche space). A final measure of aggregation is the V‐ratio, measuring associations between species. The V‐ratio for this network was significantly different from the null model, suggesting a positive aggregation of *Lasioglossum* (*Homalictus*) species within elevational niches (null = 1, observed = 8, *p* < 0.001; Table 1). Combined, our analyses suggest no competitive exclusion among bee species for niche use, but that many species still coincide in their occupancy of elevational niches.

**FIGURE 4:**
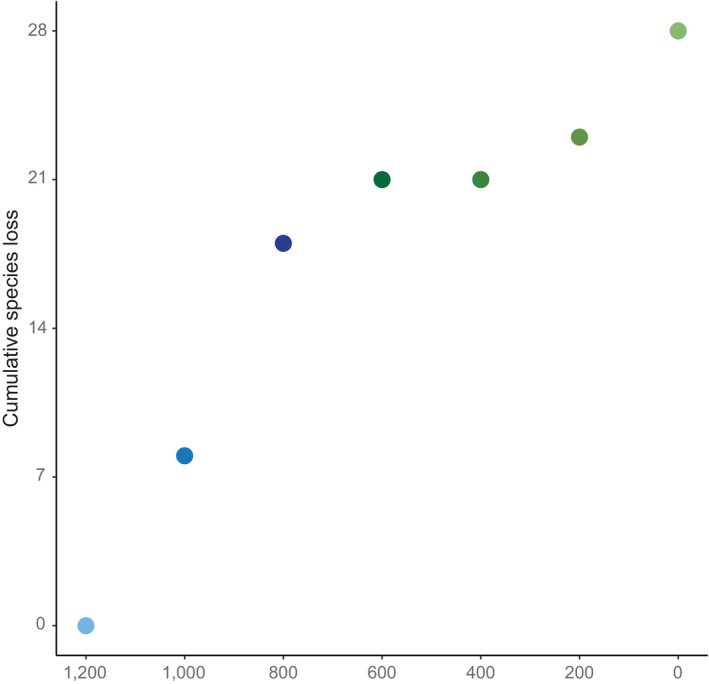
Cumulative species loss of Fijian *Lasioglossum* (*Homalictus*) bees as a function of the disappearance of their current elevational niches, starting with the highest niche (1200 m asl). Colours of the data points correspond to their location along the elevational gradient: Blue is loss of highland (> 800 m asl) elevational niches, and the green is loss of non‐highland (< 800 m asl) elevational niches.

Lastly, our estimates of the extinction slope for our occupancy data (Table 1) were significant and indicate a greater extinction vulnerability due to the loss of elevational niche than would be expected for random occupancy of elevational niche by species (null = 2.4, observed = 1.9, *p* < 0.001; Table 1). This agrees with our estimates of nestedness and NODF in that ‘loss’ of some elevational niches due to warming climates would lead to a disproportionate loss of species. We can visualise extinction vulnerabilities of the Fijian *Lasioglossum* (*Homalictus*) by graphing the cumulative loss of bee species as a function of how elevational niches may disappear under these future scenarios, assuming that the highest elevational niches would be lost first (Figure 4). The richness of *Lasioglossum* (*Homalictus*) is highest in the higher elevational niches, so loss would be greatest for elevational bands above 800 m asl. Only minimal subsequent species would be lost in the lowlands because most have already disappeared.

## 4 Discussion

We provide a complementary and statistical approach to understanding how Fijian *Lasioglossum* (*Homalictus*) communities are assembled in terms of elevational niche occupancy and potential expansion. Our results build upon prior empirical works that have demonstrated how phylogenetic conservation of elevation niche (Dorey et al. 2020) and physiological traits (da Silva et al. 2024) have forged contemporary elevational distributions. We use the inferred ancestral elevation of > 800 m asl (Dorey et al. 2020) as a proxy for a ‘mainland’ in network analyses to examine competition and extinction risks in this diverse bee clade.

### 4.1 Competition Within Elevational Bands

Our bipartite analyses indicate significant nestedness of bee species within elevational bands, with most species' diversity occurring above 800 m asl. This is concordant with Dorey et al. (2020) findings that climates currently associated with high elevations are ancestral for the Fijian *Lasioglossum (Homalictus)*, and that expansions into lower‐elevational niches are uncommon. However, our network analyses provide additional insights to those from phylogenetics (Dorey et al. 2020) and physiology (da Silva et al. 2024); in particular, our estimates of togetherness, V‐ratio and C‐score values present no evidence for exclusion in elevational niche use and instead suggest non‐competitive aggregated occupancy of elevational niches for many species. This could be directly interpreted as Fijian *Lasioglossum* (*Homalictus*) species not competing for elevational niches, but in the context of their phylogenetic history, would indicate similar patterns of elevational niche occupancy despite multiple speciation events (Dorey et al. 2020).

These network statistics can also corroborate the lack of competition for elevational niche space in the results of da Silva et al. (2024), who found no evidence of elevational competition within mountains. However, we still recommend some caution for at least two reasons: (i) within elevational bands there are non‐*Lasioglossum* (*Homalictus*) species that are still likely to compete with other bee species or pollinators for various niche dimensions (i.e., food, soil, vegetation and predator profiles) and (ii) anthropogenic effects on local ecosystems may have altered any competitive interactions that were operating prior to human colonisation of the lower elevations in the Fijian archipelago, and consequent movement into the highlands (Anderson et al. 2006). Fiji hosts an array of introduced plant species, some introduced by early Lapita and Polynesian settlements, but many more by European colonial agency (Ash 1992; Smith 1980; Thaman et al. 2012). But, the Fijian *Lasioglossum* (*Homalictus*) are super‐generalist in their floral visitations (Draper et al. 2021) and are likely resilient to such changes. Additionally, human activities have led to high levels of land slippage and vegetation clearing (e.g., Duaibe 2008; Restrepo et al. 2009), which provide the bare‐soil conditions that *Lasioglossum* prefer to nest in. Indeed, Dorey et al. (2021) showed that population sizes of the predominant lowland Fijian bee species, *L*. (*H*.) *fijiense*, increased dramatically over the last 3000 years and that this was likely due to human modifications of the environment. However, these changes are almost certain to have altered interactions with other native bees, such as the forest‐specialist *Hylaeus* (Hymenoptera: Colletidae) (Dorey et al. 2024).

The above considerations create a complex set of potential scenarios that may be very difficult to empirically interrogate. In particular, we do not understand the structure of pre‐Lapita arrival or historical post‐settlement bee‐relevant ecosystems in Fiji, but we do know that they were very different from current ecosystems (Ash 1992; Dorey et al. 2021). Due to this, it is difficult to disentangle whether current bee species assemblages reflect historical conditions or responses to extant conditions, or a combination of the two. Empirical evidence from da Silva et al. (2024) provides a physiological (mechanistic) basis for the niche conservatism that has driven past and present distributions. Warmer and drier climates at lower elevations are likely to be physiological barriers to niche breadth, as species that occupy higher elevations are less heat tolerant and desiccation resistant than species inhabiting lower elevations (da Silva et al. 2024). However, how competition (between bee species, other pollinators and predators) has influenced *Lasioglossum* (*Homalictus*) species elevational niches historically remains unknown.

### 4.2 Extinction Risks

In contrast to competition, we found very strong patterns in species occupancy of elevational niches (Figures 2 and 3) leading to very significant nestedness in species composition (Table 1). Importantly, that nestedness leads to a highly significant extinction slope (Table 1). Even if we do not fully understand how elevational niches arose for extant bee species, or if they have partitioned niches within elevational bands, our analyses indicate a worrying susceptibility of species extinction to the loss of elevational niches. Loss of an elevational niche does not mean the elevation physically disappears; rather, it means the niche is diminished or extinguished and that the bee species depending on it can no longer be sustained, or conditions change to those outside of the physiological limits of dependent bee species. This loss may occur as warming climates push cool highland niches upward into shrinking areas, eventually causing them to ‘fall’ off the top of the mountain (Chen et al. 2011; Dirnböck et al. 2011; García‐Robledo et al. 2016; Laurance et al. 2011).

Adaptation to anthropogenic climate change is possible but relies on sufficient standing genetic variation. However, the Fijian *Lasioglossum* (*Homalictus*) have diversified through niche conservatism and tracking climate up and down elevational bands (Dorey et al. 2020). Additionally, a strong phylogenetic signal in limited heat tolerance and desiccation resistance suggests that these traits are relatively constrained and limit the ability of species to rapidly adapt to changing climates (da Silva et al. 2024). Hence, any adaptations to very recent climate warming that would allow elevational niche expansion are very unlikely.

### 4.3 Intersection Between Network and Phylogenetic Analyses

There are compelling arguments provided by Marini et al. (2019) for the use of species‐habitat network analyses for managing conservation and biodiversity, but they did not explicitly consider how phylogenetics might inform those issues. However, the phylogenetic history of a species may become critical if phylogenetic inertia in the use of habitats is substantial. Conversely, while phylogenetic trait analyses are well suited to understanding how species‐specific features have evolved over time, they are not well suited for exploring how different lineages interact with each other at a landscape scale. Alternatively, network analyses can explore species interactions across niche spaces, but do not explicitly ‘build‐in’ phylogenetic histories. A key issue involves the rate of species adaptation to different niches relative to the rate at which specific niches are modified or lost. In other words, to what extent might species‐level adaptations mitigate network disruption when species differ in their potential for adaptation? That question, in turn, will depend on how species interact when colonising new niches. These questions all belong to the field of community ecology; however, advancements in phylogenomic analyses that enable more detailed assessments of species interactions across geographic space in the future are needed to enhance our ability to understand how historical species interaction networks across space have shaped present‐day patterns.

## 5 Conclusions

In this study, nestedness patterns corroborate previous phylogenetic analyses indicating higher elevation niches as ancestral to the Fijian *Lasioglossum* (*Homalictus*), with barriers to expansion into lower elevations. We provide insights into competitive exclusion, or lack thereof, within niches. We further highlight that extinction slopes indicate raised extinction risks for highland species and that loss of highland niches would result in large losses in species richness. So, in this sense, bipartite analyses of Fijian *Lasioglossum* (*Homalictus*) may provide some landscape‐level conservation insight, as the conservation of species in different elevational bands might not be affected by the packing of species into limited niche space. However, the loss of higher elevation niches will most strongly impact the Fijian *Lasioglossum* (*Homalictus*) fauna.

## Author Contributions

**Patricia S. Slattery:** conceptualization (equal), formal analysis (lead), methodology (lead), project administration (equal), visualization (lead), writing – original draft (equal), writing – review and editing (equal). **James B. Dorey:** data curation (equal), formal analysis (supporting), funding acquisition (equal), visualization (supporting), writing – review and editing (equal). **Cale S. Matthews:** writing – review and editing (equal). **Justin L. Holder:** writing – review and editing (equal). **Olivia K. Davies:** data curation (equal), writing – review and editing (equal). **Mark I. Stevens:** funding acquisition (equal), writing – original draft (equal), writing – review and editing (equal). **Michael P. Schwarz:** conceptualization (equal), funding acquisition (equal), methodology (supporting), project administration (equal), writing – original draft (equal), writing – review and editing (equal). **Carmen R. B. da Silva:** data curation (equal), funding acquisition (equal), project administration (equal), writing – original draft (equal), writing – review and editing (equal).

## Conflicts of Interest

The authors declare no conflicts of interest.

## Supporting information


**Data S1.** Nestedness matrix depicting weighted species occupancy across the elevational gradient, separated into 200 m asl elevational niches. Solid cells in the matrix denote the presence of Fijian *Lasioglossum* (*Homalictus*) species within that elevational niche, and the number depicts the number of specimens represented by that species. Colours correspond to their distribution across the elevational gradient, with blue representing highland (> 800 m asl) elevational niches, and green the non‐highland (< 800 m asl) elevational niches.

## Data Availability

Aligned sequences and collection details are available on Figshare, along with R code scripts (10.25451/flinders.26501113).
