# Supplementary material for: Network Assemblages of Elevational Niche‐Associated Diversity in Fijian Native Bees
Source: Ecol Evol. 2025 Mar 2;15(3):e71073. doi: 10.1002/ece3.71073 (PMC11873371; doi:10.1002/ece3.71073)
Supplement: Supplementary file 1 — Data S1. Nestedness matrix depicting weighted species occupancy across the elevational gradient, separated into 200 m asl elevational niches. Solid cells in the matrix denote the presence of Fijian Lasioglossum (Homalictus) species within that elevational niche, and the number depicts the number of specimens represented by that species. Colours correspond to their distribution across the elevational gradient, with blue representing highland (> 800 m asl) elevational niches, and green the non‐highland (< 800 m asl) elevational niches. [file ECE3-15-e71073-s001.pdf]

	L. fijiense	*L. tuiwawae*	*L. groomi*	*L. nadarivatu*	*L. concavum*	*L. ostridorsum*	*L. hadrandrum*	*L. atritergus*	*L.* sp. S	*L.* sp. X	*L. termile*	*L.* sp. T	*L. kaicolo*	*L.* sp. K	*L.* sp. V	*L.* sp. H	*L.* sp. O	*L.* sp. 1	*L.* sp. D	*L.* sp. G	*L. taveuni*	*L. achrostus*	*L.* sp. J	*L.* sp. F	*L.* sp. W	*L.* sp. N	*L.* sp. Y	*L.* sp. I
800 m	183	507	101	51	42		21	6		1			6	2	5				3	3			2			1	1	
0 m	404						1										1				3							1
1,000 m	81	12	7	49	45	26	23	25	37	6	10	3	1	4		4	1	2				2		1	1			
600 m	177	60	1	1	7		26										2	2										
1,200 m						54	1	17		28		5	1															
400 m	55	21					1																					
200 m	29	11	1				5																					
